# The Army’s Ten Commandments

**Published:** 1917-09

**Authors:** 


### THE ARMY'S TEN COMMANDMENTS.

1. When on guard thou shalt challenge all parties approaching thee.

2. Thou shalt not send any engraving, nor any likeness of any airship in heaven above, nor any postcard of the earth beneath, nor of any submarine in the sea, for I, the Censor am a jealous Censor, visiting iniquities of the offenders with three months' C.B., but owing mercy unto thousands by letting their letters go first of those who obey my commandments.

3. Thou shalt not use profane language unless under extraordinary circumstances, such as seeing thy mate shot or getting petrol in thy tea.

4. Remember the soldier's week consists of seven days. Six days shalt thou labor, and on the seventh thou shalt do all odd jobs.

5. Honor thy King and country; keep thy rifle well oiled, and shoot straight, that thy days may be long in the land the enemy giveth thee.

6. Thou shalt not steal thy comrade's kit.

7. Thou shalt not kill time.

8. Thou shalt not adulterate thy mess tin by using it as a shaving mug.

9. Thou shalt not bear false witness against thy comrade, but preserve silence on his out-goings and in-comings.

10. Thou shalt not covet thy Corporal's post, nor thy Sergeant's, nor thy Sergeant-Major's, but by thy duty and perseverance thou shalt rise to the position of Field Marshal—Balkan News (Circulated among the forces at Salonica.)—The Kinsman.

